# Delivery of Therapeutic AGT shRNA by PEG-Bu for Hypertension Therapy

**DOI:** 10.1371/journal.pone.0068651

**Published:** 2013-07-19

**Authors:** Yu-Qiang Wang, Fei Wang, Xiao-Qin Deng, Jing Sheng, Shu-Yan Chen, Jing Su

**Affiliations:** 1 Department of Geriatrics, Xinhua Hospital Affiliated to Shanghai Jiao Tong University School of Medicine, Shanghai, China; 2 Department of Radiation Ontology, the First Affiliated Hospital of Dalian Medical University, Dalian, China; 3 Department of Geriatrics, Shanghai Ninth People's Hospital Affiliated to Shanghai Jiao Tong University School of Medicine, Shanghai, China; 4 School of Pharmacy, Shanghai Jiao Tong University, Shanghai, China; National University of Ireland, Galway, Ireland

## Abstract

Gene silencing by RNA interference (RNAi) is a promising approach for gene therapy. However, up to today, it is still a major challenge to find safe and efficient non-viral vectors. Previously, we reported PEI-Bu, a small molecular weight PEI derivative, as an efficient non-viral carrier. However, like many PEI-based polymers, PEI-Bu was too toxic. In order to reduce cytotoxicity while maintain or even enhance transfecion efficiency, we modified PEI-Bu with poly(ethylene glycol) (PEG) to obtain PEG-Bu, and used it to delivery a theraputic short hairpin RNA (shRNA) targeting angiotensinogen (AGT) to normal rat liver cells (BRL-3A), which was a key target for the treatment of hypertension. The structure of PEG-Bu was confirmed by proton nuclear magnetic resonance (^1^H-NMR). Gel permeation chromatography (GPC) showed that the weight average molecular weight (Mw) of PEG-Bu was 5880 Da, with a polydispersity of 1.58. PEG-Bu could condense gene cargo into spherical and uniform nanoparticles with particle size (65–88 nm) and zeta potential (7.3–9.6 mV). Interestingly and importantly, PEG-Bu displayed lower cytotoxicity and enhanced tranfection efficiency than PEI-Bu after PEGylation in both normal cells BRL-3A and tumor cells HeLa. Moreover, PEG-Bu could efficiently delivery AGT shRNA to knockdown the AGT expression. To sum up, PEG-Bu would be a promising non-viral vector for delivering AGT shRNA to BRL-3A cells for hypertension therapy.

## Introduction

The renin-angiotensin system (RAS) has been proved to play a significantly important role in the pathogensis of hypertension [1]. Angiotensinogen (AGT) which is mainly produced in the liver is the source of the RAS. Previous studies have suggested a direct correlation between AGT and blood pressure. For instance, Ishigami T et al [2] found that positive correlations existed between plasma AGT concentration and systolic blood pressure (SBP) (r = 0.232, P = 0.0015), diastolic blood pressure (DBP) (r = 0.183, P = 0.0126), and elevated plasma AGT concentrations led to increased blood pressure. In addition, overexpression of the AGT gene in transgenic mice generated by injecting the entire rat angiotensinogen gene into the germline of NMRI mice led to the development of hypertension [3]. On the contrary, AGT gene knockout mice did not generated AGT and resulted in reduction of blood pressure [4]. Therefore, suppressing of AGT expression may be a new strategy for the treatment of hypertension.

Gene silencing by RNA interference (RNAi) is a promising approach in the area of gene therapy for the treatment of many genetic and acquired diseases [5], [6]. RNAi is the process of suppressing the expression of a protein by inducing the degradation of specific mRNA sequences. The safe and efficient delivery of therapeutic siRNA or shRNA to the target site is the prerequisite for successful gene therapy. Gene delivery vectors are classified into two categories: viral and non-viral vectors. In recent years, non-viral vectors have been investigated intensively for gene delivery due to their ease of production, safety, lower immune response, the ease of chemical modification and the ability to transfer larger pDNA molecules [7], [8]. Among the vast family of non-viral vectors, the cationic polyethylenimine (PEI) has taken a prominent position. PEI could effectively condense nucleic acids into nanoparticles and be capable of transfecting a variety of cell lines both in vitro and in vivo [9].

In our previous work, we reported PEI-Bu, a biscarbamate cross-linked low molecular weight PEI derivative, as an efficient carrier in comparison to commercially available PEI 25 kDa, which was considered as the most popular “gold standard” in gene transfection. However, PEI-based polymers had a trait in common: they were too toxic [10], and PEI-Bu was no exception. For example, the cell viability was just below 50% when the concentration exceeded 30 μg/mL. High cytotoxicity will be a severe barrier for its further application. In addition, PEI/DNA complexes tended to aggregate under physiological conditions, which was not appropriate for gene transfer. Therefore, PEI has to be modified to reduce cytotoxicity while maintain or even enhance efficiency. Among so many modifiers, PEG was wildly used due to its safety and water solubility. What is more, chemical modification of PEI with PEG could provide polymer with improved biocompatibility, lower the cytotoxicity of PEI, facilitate the formation of complexes with diminished aggregation, protect complexes from macrophage uptake, reduce non-specific interactions with serum proteins in bloodstream, and prolong circulation time of complexes in vivo [11], [12]. Based on this concept, in the present study, we modified PEI-Bu with PEG to obtain PEG-Bu. We determined the characterization of PEG-Bu, the characterization of PEG-Bu/pDNA complexes and the influence of PEGylation on cytotoxicity and transfection efficiency in both normal cells BRL-3A and tumor cells HeLa. At last, we determined its ability to delivery a theraputic AGT shRNA to BRL-3A cells, one of the optimal therapeutic targets for hypertension therapy, and observed the gene silencing effect.

## Materials and Methods

### Materials

Branched PEI (800 Da, 25 kDa), Ethidium bromide (EB), and 3-(4,5-dimethylthiazol-2-yl)-2,5-diphenyltetrazoliumbromide (MTT) were sourced from Sigma-Aldrich (St Louis, MO, USA). The Methoxy- poly (ethylene glycol)-Succinimidyl Carbonate (mPEG-sc; Mw  = 2000 Da) was purchased from Yarebio Cooperation (Shanghai, China). PEI-Bu was synthesized in our previous work. Luciferase assay kit was purchased from Promega (Madison, WI, USA). MicroBCA protein assay kit was purchased from Pierce (Rockford, IL, USA). All other chemicals used were of analytical grade.

Dulbecco's modified Eagle medium (DMEM), Trypsin-EDTA and fetal bovine serum (FBS) were obtained from PAA (colbe, Germany). GFP bearing AGT shRNA was purchased from JIKAI Cooperation (Shanghai, China). The pDNA was pGL3-Control (Promega, Madison, WI, USA) encoding firefly luciferase.

### Synthesis of PEG-Bu

To synthesize PEG-Bu, 0.06 mmol of PEI-Bu, which was synthesized according to our previous study [13], was dissolved in 0.1 M sodium bicarbonate, then 0.02 mmol of mPEG-Sc was added to PEI-Bu with stirring. The reaction was carried out for 4 h at room temperature. The sample was subsequently dialyzed against distilled water in a dialysis tube (MWCO: 3500 Da) for 2 days and lyophilized to yield the resultant polymer PEG-Bu. The PEG-Bu was stored at −20°C for further use.

### Characterization of PEG-Bu

PEG-Bu was estimated by measuring ^1^H nuclear magnetic resonance (^1^H-NMR) (Mercury plus 400, Varian Inc). GPC relative to PEG standards (molecular weight range: 106, 430, 633, 1400, 4290, 7130, 12,600, 20,600 Da) was used to measure the molecular weight of PEG-Bu, using a Waters high-pressure liquid chromatography (HPLC) system (Milford, MA)with ultrapure water as the mobile phase.

### Preparation and characterization of PEG-Bu/pDNA complexes

PEG-Bu/pDNA complexes were prepared by adding PEG-Bu solution to pDNA solution at the desired weight ratio. The formulation and stability of complexes were evaluated by agarose gel electrophoresis. The complexes were placed on 1% (w/v) agarose gels containing 0.5 μg/mL ethidium bromide in Tris-acetate (TAE) buffer. The particle size and zeta potential of PEG-Bu/pDNA complexes in ultrapure water were examined by a particle size analyzer (90Plus; Brookhaven, Holtsville, NY). The morphology of PEG-Bu/pDNA complexes at w/w 5 was observed with atomic force microscopy (AFM) (E-Sweep; SII Nanotechnology, Inc, Chiba, Japan).

### Cytotoxicity assay

Cytotoxicity profiles of PEG-Bu and PGE-Bu/pDNA complexes were examined by MTT assay in BRL-3A and HeLa cell lines according to a method previously reported [14]. The dose of gene cargo used in the PGE-Bu/pDNA cytotoxicity experiments was 200ng pDNA/well in 96-well plates.

### Luciferase activity assay and flourescence-activated cell sorting analysis (FACS)

Transfection efficiency of PEG-Bu in terms of luciferase activity and FACS were determined according to a method previously reported [14]. The dose of gene cargo used in the transfection experiments was 500 ng pDNA/well in 48-well plates(Luciferase activity assay), and 2 μg shRNA/well in 6-well plates (FACS).

### Real-time PCR analysis

After transfection for 48 h, total RNA was extracted from BRL-3A cells using Trizol reagent according to the manufacturer’s instructions. 2μL of cDNA were subjected to PCR reactions using specific primers. Real-time PCR was performed using MA3000P thermal cycler (Applied Biosystems). The conditions for PCR were as follows: 30 cycles including 95°C for 30 seconds, 60°C for 30 seconds, and 72°C for 45 seconds. The house keeping gene β-actin was used as an internal reference. Sequences of the AGT primers were as follows: forward, 5′-CATCTTCCCTCGCTCTCTG-3′ and reverse, 5′-GCCTCTCATCTTCCCTTGG-3′. Sequences of the β-actin primers were as follows: forward, 5′-CTGTCCCTGTATGCCTCTG-3′ and reverse, 5′-TGTCACGCACGATTTCC-3′. mRNA level of AGT was normalized to that of β-actin.

### Western blot analysis

After transfection for 72h, total protein was extracted from BRL-3A cells according to the protocols of the total cellular soluble protein preparation kit (GenMed Scientifics Inc, USA). Protein concentrations were examined by the BCA-100 Protein Quantitative Analysis Kit (Shenergy Biocolor, Shanghai, China). 30 μg protein was separated on 8% SDS-PAGE gels and transferred to nitrocellulose membranes (Whatman, Germany) by electroblotting. Membranes were blocked in 5% nonfat dry milk in TBST buffer for 2 h at room temperature and incubated with mouse anti-AGT antibodies (1:1000, Bender, Austria) overnight at 4°C. After three washes in TBST, membranes were incubated with Goat Anti-Mouse IgG (1:8000, Jackson, MI, USA) for 1 hour at room temperature, then the membranes were incubated with Enhanced Chemiluminescence Detection Reagent for 3 min. The protein bands were visualized after exposition with x-ray film in an imaging cassette. The housekeeping gene β-actin was used as control. Images were quantitated with Quantity One Software.

### Statistical analysis

Data were shown as mean±standard deviation. Statistical analysis was performed with SPSS software (v 19.0; SPSS Inc, Chicago, IL). The significance of the differences between two groups was determined by Student's t-test. P<0.05 was considered to be statistically significant.

## Results and Discussion

### Synthesis and characterization of PEG-Bu

**Figure 1:**
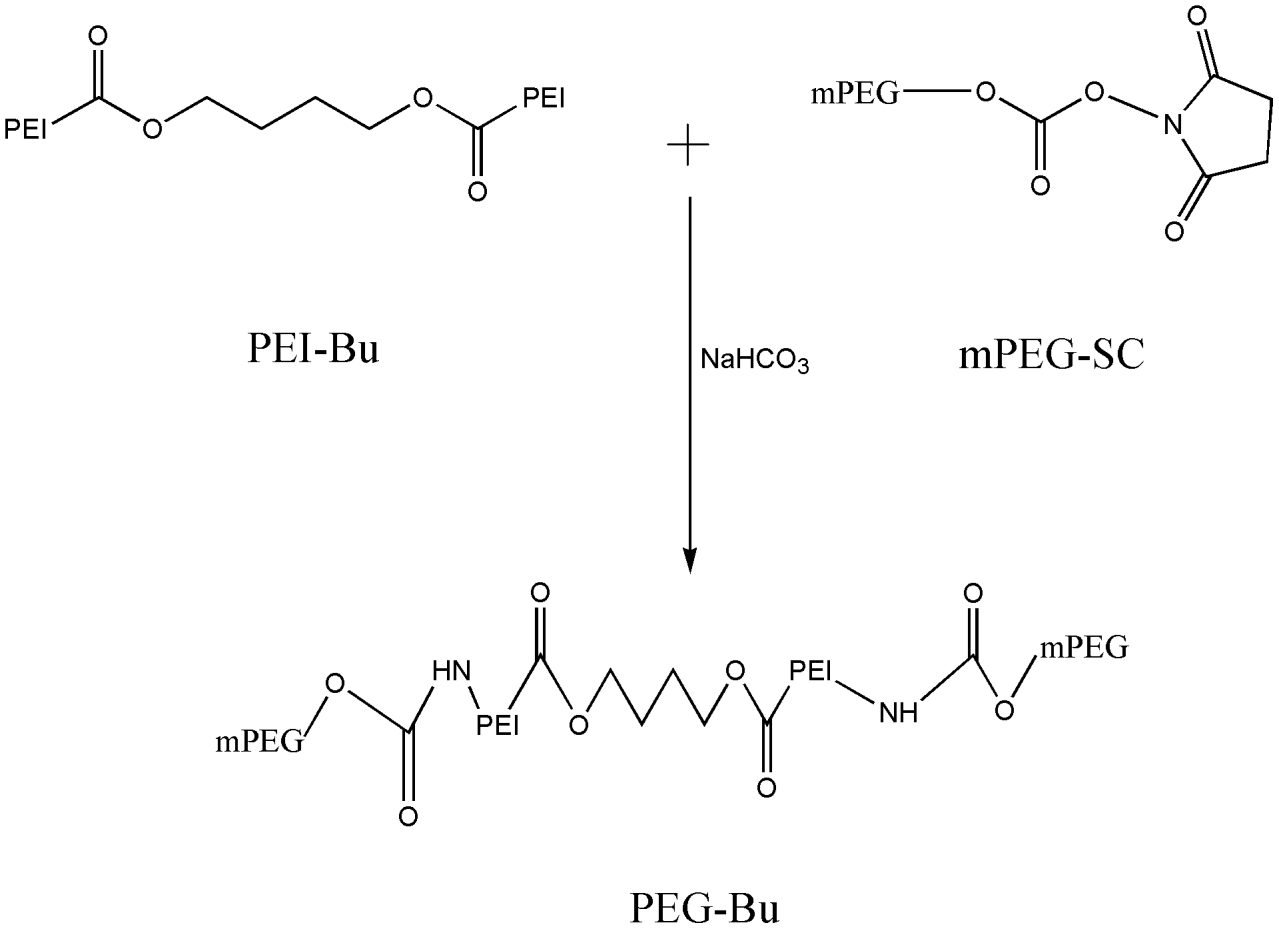
Reaction scheme of PEG-Bu.

**Figure 2:**
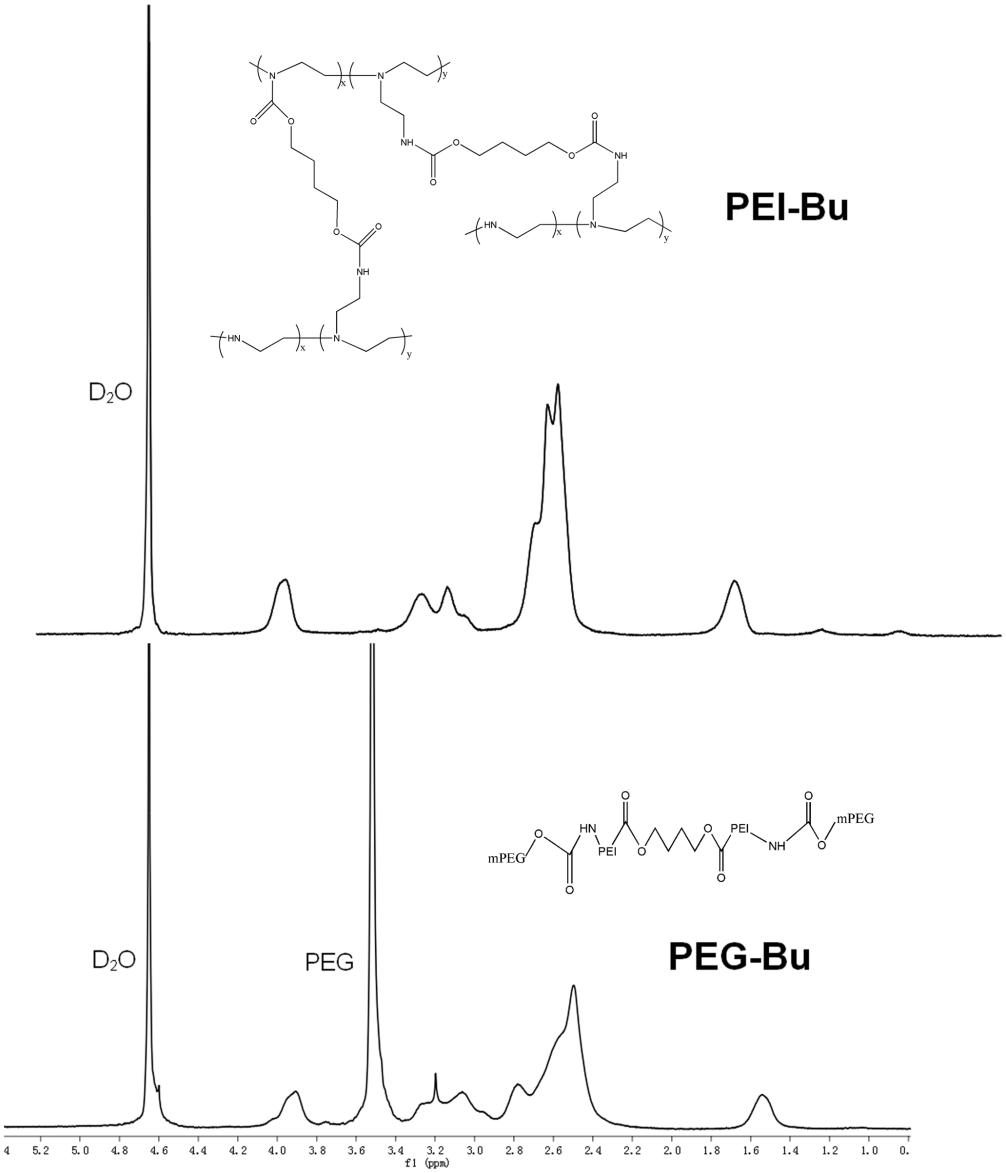
Representative ^1^H-NMR spectra of PEG-Bu.

Figure 1 illustrated the reaction scheme of PEG-Bu. PEG-Bu was synthesized through the conjugation of mPEG-Sc to PEI-Bu. The structure of PEG-Bu was estimated by measuring ^1^H-NMR. As shown in Figure 2, the proton peak appeared at 3.5 ppm in the PEG-Bu attributed to PEG (–OCH_2_CH_2_–), indicating that PEG was successfully conjugated to the PEI-Bu chain. Table 1 showed the molecular weight of PEG-Bu. The weight average molecular weight (Mw) of PEG-Bu measured by GPC was 5880 Da, with a polydispersity of 1.58. These results indicated that PEG-Bu was successfully synthesized.

**Table 1:** The weight average molecular weight (Mw), number average molecular weight (Mn), and molecular weight distribution (Mw/Mn) of the polymers.

Sample	Mw	Mn	Mw/Mn
PEI-Bu	4289	3278	1.31
PEG-Bu	5880	3714	1.58

### Characterization of PEG-Bu/pDNA Complexes

**Figure 3:**
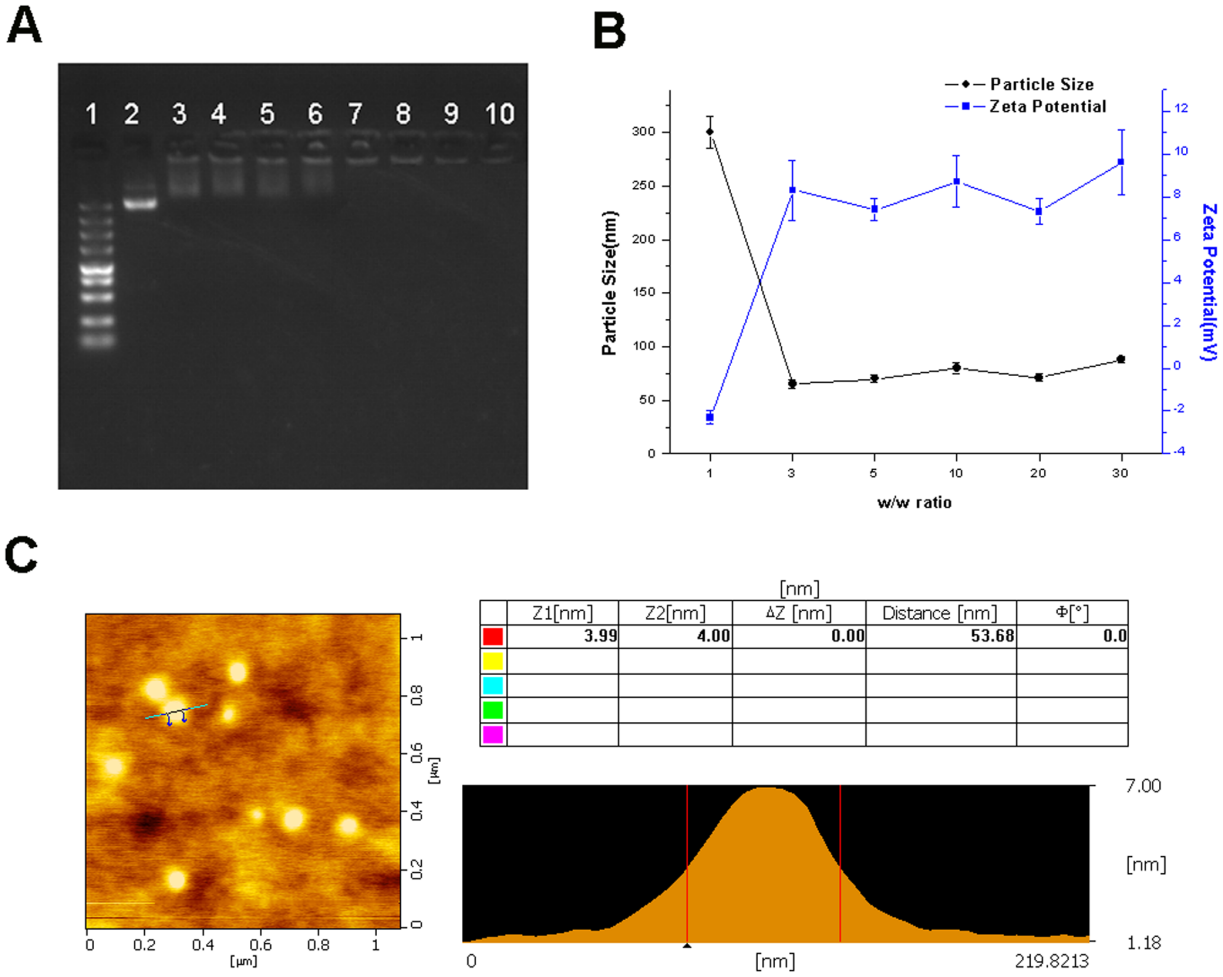
Characterization of PEG-Bu/pDNA complexes. A: Agarose gel electrophoresis of PEG-Bu/pDNA complexes at various w/w ratios. 1: Marker; 2: naked pDNA; 3–10: polymer/pDNA complexes at w/w ratios of 0.1,0.3,0.5,1,3,5,10,20. B: Particle size and zeta potential of PEG-Bu/pDNA complexes at various w/w ratios. C: Representative atomic force microscopic (AFM) image of PEG-Bu/pDNA complexes at a w/w ratio of 5.

Gene cargo should be condensed into stable nanoparticle for efficient delivery. Agarose gel electrophoresis was performed to measure the pDNA condensation ability of PEG-Bu. Naked pDNA was used as the control. As displayed in Figure 3A, PEG-Bu could completely retard the migration of pDNA when the w/w ratio was 3, indicating that PEG-Bu/pDNA complexes were completely formed at this w/w ratio. This phenomenon could be explained that the positive charges of PEG-Bu were able to neutralize the negative charges of the phosphate groups in pDNA, thus retarding the pDNA migration. The formation of polymer/pDNA was a necessary step in gene delivery, and this could protect the gene cargo from enzymatic degradation [15], thus prolonging the half-life of gene cargo.

As shown in Figure 3B, the particle size and zeta potential of PEG-Bu/pDNA complexes varied at different w/w rations. At w/w 1, the particle size was 300 nm. The particle size reached to 65 nm at w/w 3, and then kept relatively constant between 65 nm and 88 nm with polydispersity ranging 0.170–0.338. This size was appropriate for cellular uptake, especially for in vivo hepatocyte transfer, because the majority of fenestrate of the liver sinusoid was smaller than 200 nm in diameter [16]. As for the zeta potential, it was negative when the w/w was 1. However, the zeta potential became positive and ranged from 7.3mV to 9.6mV when the w\w exceeded 3. We have previously examined the zeta potential of PEI-Bu, and the data was around 20 mV [13]. The reduced zeta potential of PEG-Bu could be ascribed to the shielding effect on positive charge of the complexes by the PEG chains [17].

The representative morphologies of PEG-Bu/pDNA complexes (w/w 5) under AFM were shown in Figure 3C. The PEG-Bu/pDNA complexes were spherical in shape with compact structure. Also, the diameters of the complexes were between 45 nm and 55 nm, which was consistent with the result determined by DLS.

### Cytotoxicity assay

**Figure 4:**
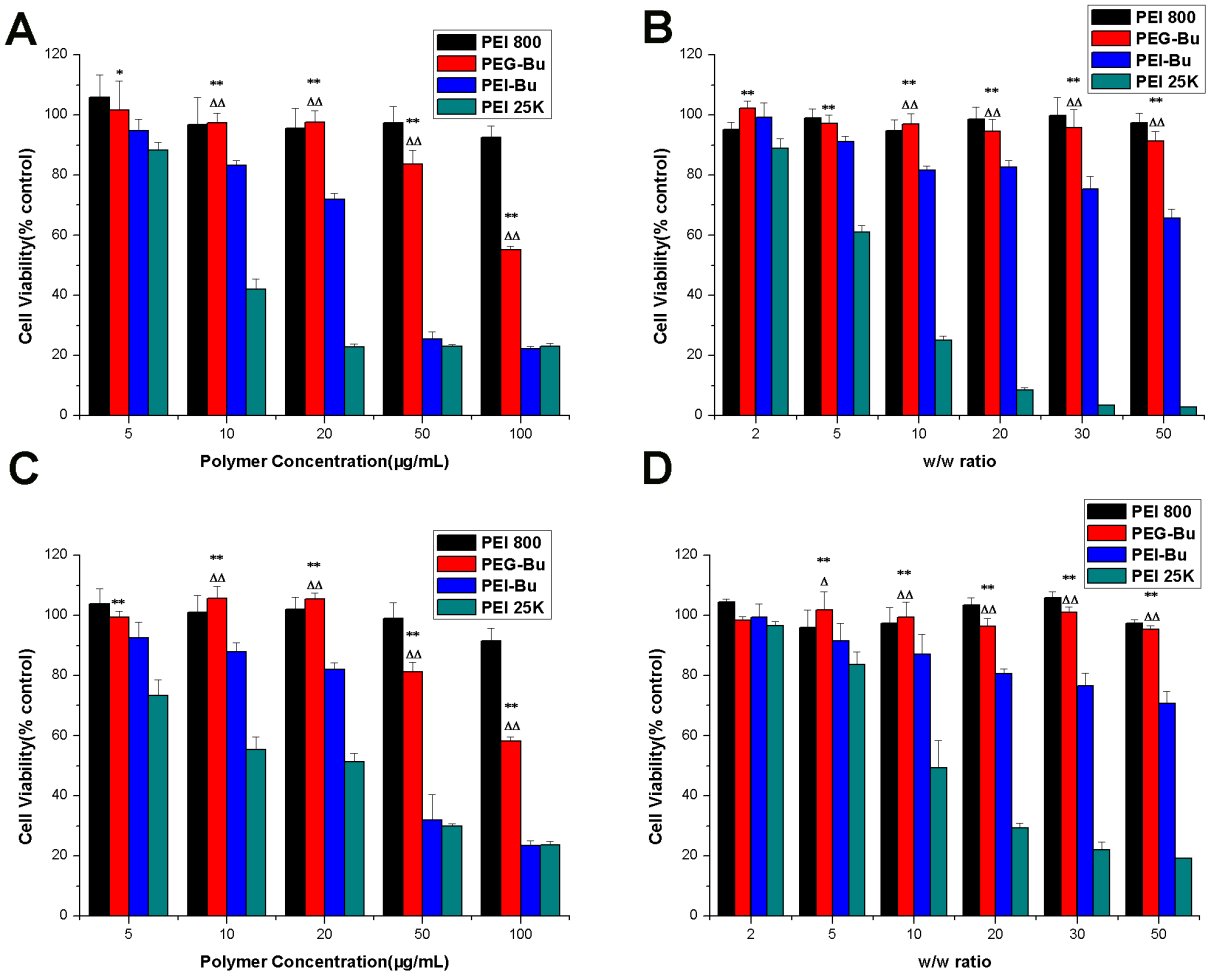
Cytotoxicity of PEG-Bu. Cytotoxicity of PEG-Bu at various concentrations and cytotoxicity of PEG-Bu/pDNA complexes at various w/w ratios in BRL-3A (A, B) and HeLa (C, D) cell lines. (n = 5, error bars represent standard deviation, *p<0.05, **p<0.01 vs PEI 25 kDa, ^Δ^p<0.05, ^ΔΔ^p<0.01 vs PEI-Bu).

The cytotoxicity of a carrier is a significantly important factor determining whether it can be used in clinical applications. PEG-Bu could produce immediate cytotoxicity mediated by free PEG-Bu and delayed cytotoxicity mediated by PEG-Bu/pDNA complexes. Therefore, the two types of cytotoxicity were all analyzed by MTT assay in BRL-3A and HeLa cell lines. As summarized in Figure 4 (A, C), the cell viability of PEG-Bu, PEI-Bu and PEI 25 kDa displayed a decreasing trend with increasing polymer concentration, indicating that the cytotoxicity of these polymers was concentration-dependent. PEG-Bu showed very small or negligible cytotoxicity when the concentrations were below 50 μg/mL. For example, the cell viabilities were 101%±9% and 99%±2% at a polymer concentration of 5 μg/mL in BRL-3A and HeLa cells, respectively. And the data slightly decreased to 84%±4% and 81%±3% respectively with PEG-Bu concentration increasing to 50 μg/mL. However, the cytotoxicity of PEG-Bu increased drastically at concentrations over 50 μg/mL. Moreover, the cytotoxicity of PEG-Bu was much lower than that observed with PEI-Bu and PEI 25 kDa at the same concentration (P<0.05) in both of the two cells. As for the cytotoxicity of polymer/pDNA complexes, PEG-Bu/pDNA complexes also exhibited lower cytotoxicity than PEI-Bu/pDNA and PEI 25 kDa/pDNA complexes. In addition, both PEI 800 Da and PEI 800 Da/pDNA complexes displayed negligible cytotoxicity, and the cell viabilities were over 90% at the tested concentrations or w/w ratios. Surface charge sourced from the PEI segment was one of critical factors influencing the cytotoxicity of PEI-based polymers [17]. PEG modification could help reduce the number of PEI amino groups by coupling reaction, thus reducing the surface charge [18]. Therefore, PEG-Bu with lower cytotoxicity than PEI-Bu was probably due to the reduction in positive charge through incorporation of PEG. In addition, the cytotoxicity of PEG-Bu was lower than that of PEI 25 kDa was possibly ascribed to the lower molecular weight of PGE-Bu (Mw: 5880 Da), because PEI with low molecular weight induced lower cytotoxicity than PEI with high molecular weight [19], [20].

### Transfection experiments in vitro

**Figure 5:**
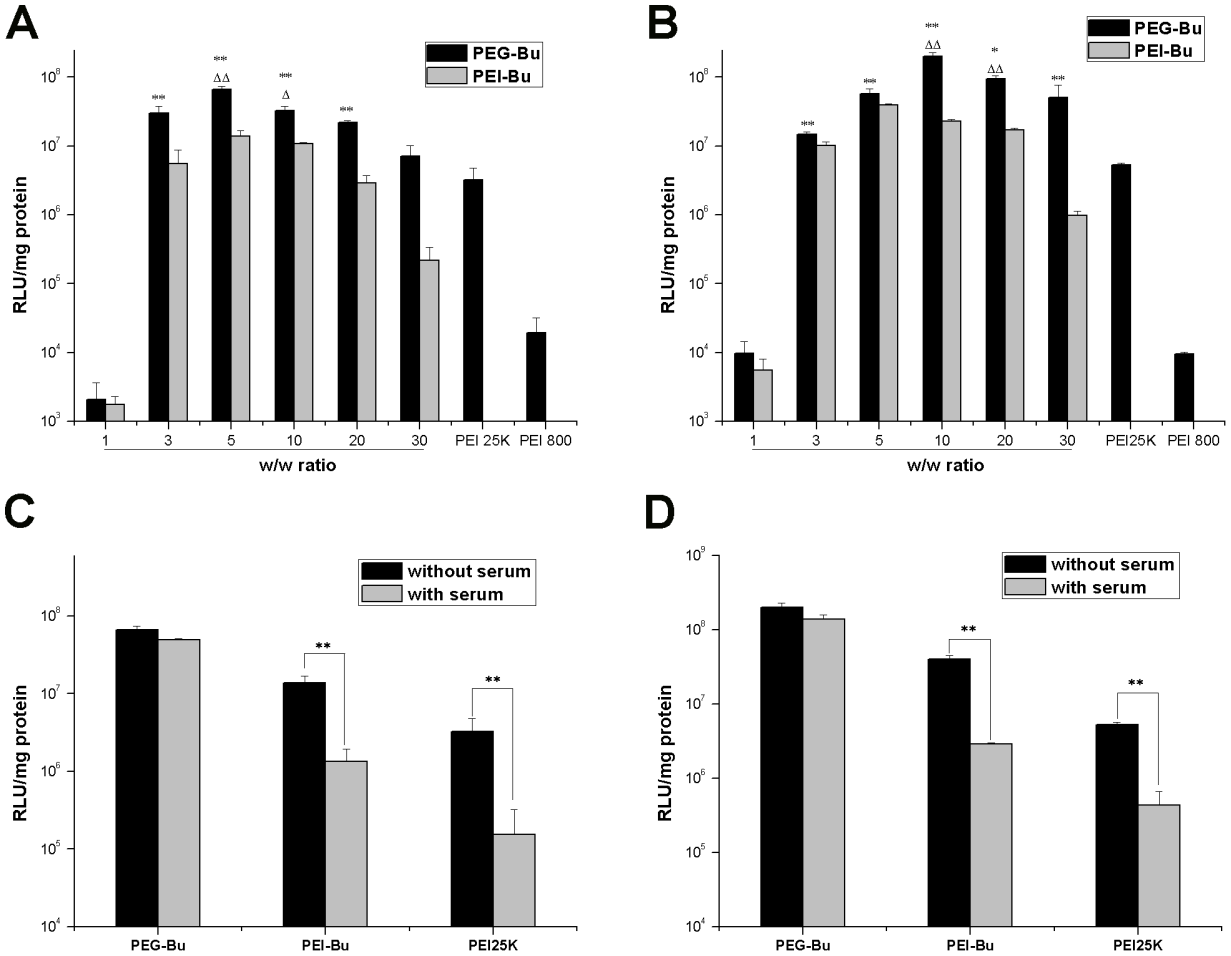
Transfection efficiency of PEG-Bu. Transfection efficiency of PEG-Bu/pGL3-Control complexes at various w/w ratios in serum–free media^,^ and transfection efficiency of PEG-Bu/pGL3-Control complexes (at optimal w/w) in serum-containing (10%) media in (A, C) BRL-3A, and (B, D) HeLa cell lines (n = 3, error bars represent standard deviation. As for A and B, *p<0.05, **p<0.01 vs PEI 25K, ^Δ^p<0.05, ^ΔΔ^p<0.01 vs PEI-Bu at optimal w/w 5).

The transfection efficiency of PEG-Bu/pDNA at various w/w ratios was observed in BRL-3A and HeLa cells, using pGL3-control as the reporter gene. PEI 25kD and PEI 800 Da at optimal w/w ratio 2 were used as positive controls. As shown in Figure 5 (A, B), transfection efficiency of PEG-Bu appeared different at various w/w ratios, and the efficiency increased with increasing w/w ratios followed by a decreasing trend. The possible explanation for this phenomenon may be as follows: at low w/w ratios, the complexes were unstable, thus producing low transfection efficiency. On the contrary, at high w/w ratios, the complexes were too stable to release the DNA from the complexes, which resulted in low efficiency [14].

The highest transfection activity of PEG-Bu was obtained at w/w 5 and 10 in BRL-3A and HeLa cells, respectively. And PEI-Bu displayed the highest efficiency at w/w 5, irrespective of the cell lines. PEG-Bu/pDNA complexes at w/w 5 showed a 4.8-fold higher transfection efficiency in comparison to PEI-Bu at its optimal w/w 5 (p<0.01), and 20.6-fold higher transfection efficiency than PEI 25 kDa (p<0.01) in BRL-3A cells. In HeLa cells, PEG-Bu/pDNA complexes at w/w 10 resulted in a 5.0-fold higher transfection than PEI-Bu at its optimal w/w 5((p<0.01), and 37.6-fold higher transfection efficiency than PEI 25 kDa (p<0.01). Moreover, PEG-Bu exhibited higher transfection efficiency than PEI-Bu(w/w 5) at w/w ranging 5–10 and 10–20 in BRL-3A and HeLa cells, respectively (p<0.05). At w/w ranging 3–30, PEI-Bu showed much higher transfection efficiency than PEI 800 Da (p<0.01), which was in agreement with a previous report [13].

As described above, the transfection efficiency of PEG-Bu was improved after PEGylation in two different cells, although several previous studies reported that modification of cationic polymers with PEG could lead to a considerable reduction in transfection efficiency because of an inefficient cellular uptake [21], [22]. The enchanced transfection of PEG-Bu than PEI-Bu was probably due to its low cytotoxicity, which was in agreement with a study Kissel et al [23] previously reported. Kissel conjugated PEI with different PEGs and found a correlation between cytotoxicity and transfection, that was, polyplexes with lower cytotoxicity displayed higher transfection efficiency.

As for PEG-Bu, a cationic polymer, it was a major hurdle for in vivo application that the positively charged polymer/pDNA or polymer/RNA complexes would bind with the negatively charged serum proteins, which could reduce the cellular uptake of complexes [24]. To observe the effect of serum on tansfection of PEG-Bu, transfection assays were performed in media with or without serum in BRL-3A and HeLa cells. As illustrated in Figure 5 (C, D), tansfection efficiency of PEI-Bu and PEI 25 kDa was remarkably attenuated (p<0.01) in the presence of serum, whereas transfection efficiency of PEG-Bu only slightly decreased when serum was present in the media and there were no statistically significant differences. This result suggested that transfection efficiency of PEG-Bu was not influenced by the presence of serum in the media. Previously, Kuo et al [25] reported that hydrophilic group could inhibit interaction between polymer/DNA complexes and serum protein, which would facilitate gene delivery. Therefore, for this reason, we speculated that it was the hydrophilic group PEG in PEG-Bu that maintained the high transfection efficiency in the presence of serum.

**Figure 6:**
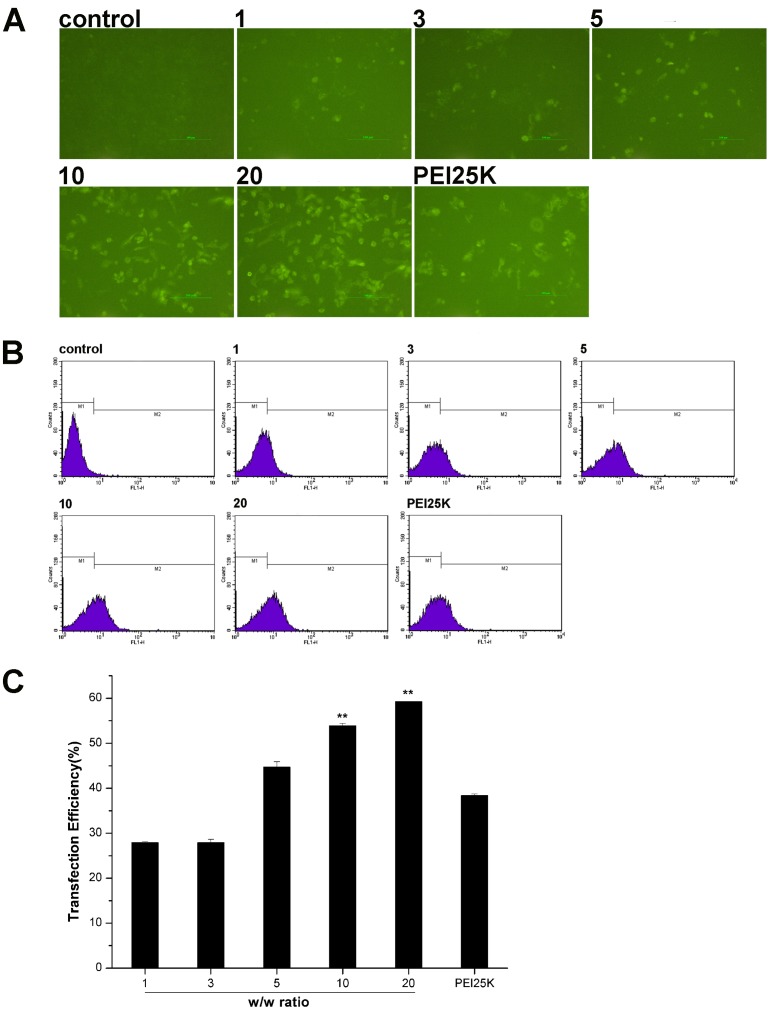
Flow cytometry assay. Transfection efficiency of PEG-Bu measured with flow cytometry in BRL-3A cells. A: fluorescence images observed with fluorescence microscopy. B: Representative histograms obtained from the flow cytometry, C: quantitative analysis of transfection efficiency of PEG-Bu as a percentage of GFP positive cells per total amount of BRL-3A cells in serum–free media. (n = 3, error bars represent standard deviation, **P<0.01 vs PEI 25K). Note: PEI 25K: PEI 25 kDa.

To obtain the optimal w/w ratio of PEG-Bu/shRNA, transfection efficiency of PEG-Bu/shRNA complexes was observed with fluorescence microscope and flow cytometry in BRL-3A cells, using GFP bearing shRNA to form PEG-Bu/shRNA complexes. Cells transfected with PBS were used as control, and the autofluorescence of these cells was excluded from analysis. Figure 6A directly visualized the gene expression of GFP in BRL-3A cells, which suggested that shRNA was successfully transported into the cells. In addition, PEG-Bu/shRNA displayed the most bright fluorescent spots at a w/w ratio of 20. As illustrated in Figure 6C, the transfection efficiency of PEG-Bu increased with increasing w/w ratios in BRL-3A cells, and the highest efficiency (59%±0.1%) was obtained at w/w 20, which was in agreement with the results of the fluorescence images. Moreover, at w/w ratios from 10 to 20, PEG-Bu displayed higher efficiency (54%±0.5%–59%±0.1%) than PEI 25 kDa (38%±0.3%) (p<0.01).

### Gene silencing effect of AGT shRNA

**Figure 7:**
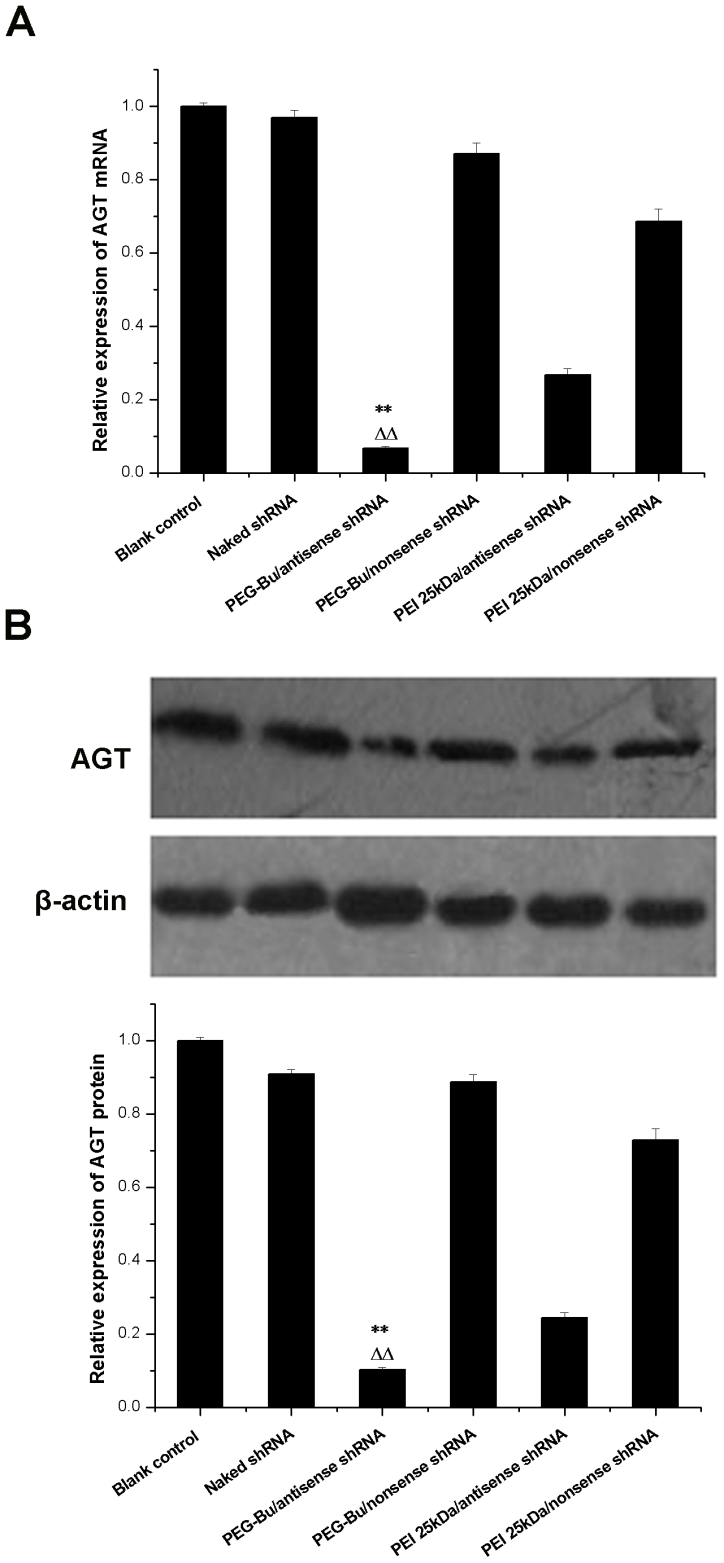
Gene silencing effect of PEG-Bu/shRNA (w/w 20) in BRL-3A cell lines. A: Real-time PCR analysis of the effect of knockdown AGT expression. B: Western blot analysis of the effect of knockdown AGT expression. (**p<0.01 vs Blank control, ^ΔΔ^p<0.01 vs PEI 25 kDa/antisense shRNA).

Gene silencing by RNAi could be mediated by transfecting synthetic siRNA or shRNA. shRNA was used in this study because it would yield more durable gene silencing while transient knockdown effect was obtained when using synthetic siRNA [26], [27]. PEG-Bu/shRNA at w/w 20 was selected for transfection of BRL-3A cells. The ability of shRNA to reduce AGT gene expression in BRL-3A cells was measured by real-time RCR as well as western blot analysis. As shown in Figure 7A, the mRNA expression of AGT was down-regulated to 6.7%±0.5% and 26.8%±1.6% by PEG-Bu/antisense shRNA and PEI 25 kDa/antisense shRNA respectively. Naked shRNA produced no obvious silencing effect on the expression of AGT, indicating that shRNA could not silence expression of the target gene without carrier. As for the PEI25 kDa/nonsense and PEG-Bu/nonsense group, the mRNA expression of AGT was also down-regulated, however, this phenomenon was more obvious in the PEI25 kDa/nonsense than in PEG-Bu/nonsense group. This was probably due to the higher cytotoxicity of PEI 25 kDa than PEG-Bu, because toxic effects could impair cell function, thus giving rise to decreased AGT expression. To further confirm the gene silencing effect of shRNA in protein level, western blot analysis was performed. The expression of AGT protein was decreased to 10.3%±0.5% and 24.4%±1.5% by PEG-Bu/antisense shRNA and PEI 25 kDa/antisense shRNA, respectively. The result was in agreement with that of real time PCR, which implied that PEG-Bu could delivery AGT shRNA to efficiently suppress the expression of AGT expression, and the gene silencing effect of shRNA was obviously better when using PEG-Bu as a carrier than using PEI 25 kDa.

## Conclusion

In the present study, PEG-Bu was successfully prepared as a non-viral vector through modification of PEI-Bu with PEG. PEG-Bu could condense gene cargo into nanoparticles with suitable physicochemical properties. Interestingly and importantly, after PEGylation, PEG-Bu exhibited lower cytotoxicity and enhanced tranfection than PEI-Bu in both normal cells BRL-3A and tumor cells HeLa. In addition, PEG-Bu could delivery AGT shRNA to efficiently inhibit the expression of AGT in BRL-3A cells, which was a key target for the treatment of hypertension. Therefore, PEG-Bu would be a promising non-viral vector for delivering AGT shRNA to BRL-3A cells for hypertension therapy. Further study can be performed to investigate the in vivo RNAi effect using related PEG-Bu/shRNA complexes, or targeted gene delivery through further modification of PEG-Bu with proper targeting group.
